# Supplementary material for: Isolated Variable Domains of an Antibody Can Assemble on Blood Coagulation Factor VIII into a Functional Fv-like Complex
Source: Int J Mol Sci. 2022 Jul 23;23(15):8134. doi: 10.3390/ijms23158134 (PMC9330781; doi:10.3390/ijms23158134)
Supplement: Supplementary file 1 [file ijms-23-08134-s001.zip › ijms-1823474-supplementary.pdf]

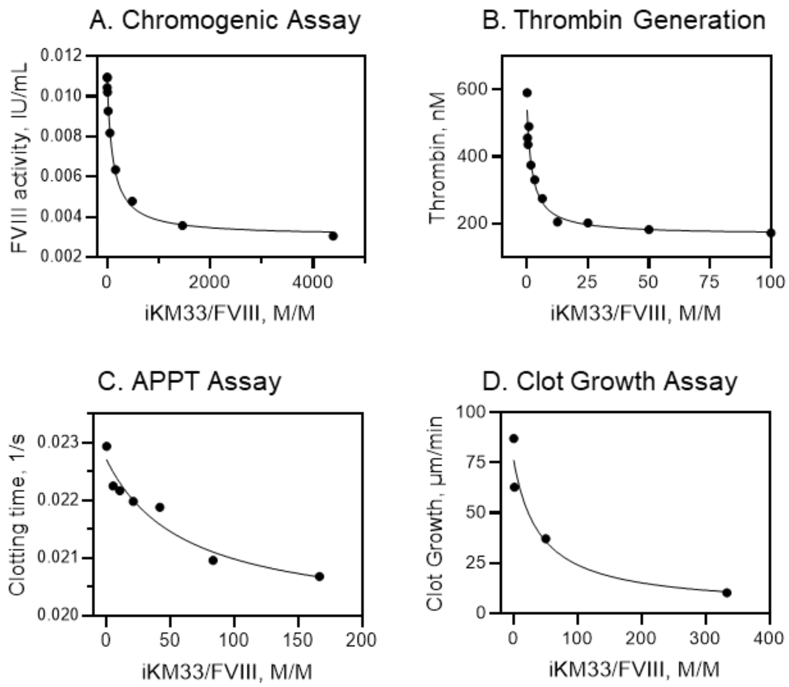

Figure S1. Effect of iKM33 on FVIII activity by different assays. FVIII (1 IU/mL) was incubated with serially diluted iKM33 for 30 min in FVIII-deficient plasma. Upon the incubation, FVIII activity was measured by Chromogenic Assay (**A**), Thrombin Generation (**B**), Activated Partial Thromboplastin Clotting Time (APPT) (**C**), and video microscopy of clot growth (**D**) assays.
